# Conductive Bicomponent Fibers Containing Polyaniline Produced via Side-by-Side Electrospinning

**DOI:** 10.3390/polym11060954

**Published:** 2019-06-01

**Authors:** Wangcheng Liu, Jinwen Zhang, Hang Liu

**Affiliations:** 1 School of Mechanical and Materials Engineering; Washington State University, Pullman, WA 99164, USA; Wangcheng.liu@wsu.edu; 2 Composite Materials and Engineering Center; Washington State University, Pullman, WA 99164, USA; jinwen.zhang@wsu.edu; 3 Department of Apparel, Merchandising, Design and Textiles; Washington State University, Pullman, WA 99164, USA

**Keywords:** polyaniline, electrospun nanofiber, conductive, side-by-side fiber

## Abstract

In this study, using a barbed Y-connector as the spinneret, camphoric acid (CSA) doped polyaniline (PANI) and polyethylene oxide (PEO) were electrospun into side-by-side bicomponent fibers. Fiber mats obtained from this side-by-side spinneret were compared with those mats electrospun from blended PEO and PANI in terms of fiber morphology, electrical conductivity, thermal stability, mechanical properties, and relative resistivity under tensile strain. The influence of different content ratio of insulating PEO (3/4/5 w/v% to solvent) and conductive PANI-CSA (1.5/2.5/3.5 w/v% to solvent) on the abovementioned properties was studied as well. Results showed that this side-by-side spinning was capable of overcoming the poor spinnability of PANI to produce fibers with PEO carrying PANI on the surface of the bicomponent fibers, which demonstrated higher electrical conductivity than blends. Although the addition of PANI deteriorated mechanical properties for both side-by-side and blended fibers when compared to the pure PEO fibers, the side-by-side fibers showed much better fiber strength and elongation than blends. In addition, the superior ductility and decent relative electrical resistivity of the side-by-side fibers imparted them great potential for flexible sensor applications.

## 1. Introduction

Smart textiles that can sense and react to an environmental stimulus, such as electricity, light, heat, mechanical pressure/strain, and chemical and biological agents, have great potential to expand the traditional ways that users interact with textiles. Among various smart textiles, wearable devices that sense, collect, process, store and display information, and transmit signals is a fast-growing area that can find broad range of applications in medical/health, sports, and personal protective apparatus for military and first response workers [1,2]. Thus, new textile materials with electrical conductivity have drawn great attention in the past decade because electrical conductivity is essential for building basic electronic devices and for connecting different units in the smart system [3]. Additionally, conductivity change resulted from the reaction between a stimulus and the conductive material leads to current change in a closed circuit and this is an important mechanism for sensing. Tradition polymers are insulative with conductivity typically lower than 10^-9^ S/cm. Therefore, developing conductive textile fibers has been an interesting area for researchers. Enormous amount of research has been carried out in developing fiber composites that are conductive by using fillers, such as carbon nanotubes, graphite and graphene, carbon black, and metal nanowires, etc. [3,4].

The discovery of intrinsically conductive polymers (ICPs) and especially the recent research endeavors showing the promise of ICPs being processed into conductive continuous fibers have opened up the playground for a new generation of smart wearables that are entirely made of polymers [1,3,4,5]. Polyaniline (PANI) is one of the conjugated ICPs that are most intensively studied because of its many advantageous properties, including low cost of the aniline monomer, easy synthesis, biocompatibility, environmental stability, and tunable conductivity [6,7,8,9]. Pristine PANI (emeraldine base) is insulating and oxidizing/doping its π-bond backbone through chemical or electrochemical methods transforms PANI into emeraldine salt, which is conductive. The process is reversible. This unique property along with its rich redox chemistry for chemical modifications imparts PANI great potential for applications in protective apparatus to sense gas vapors, biological and chemical agents, and to be used as electrostatic dissipation and electromagnetic interference shielding [9,10]. 

The aromatic conjugated double bonds on its long carbon backbone make PANI rigid compared to most conventional textile polymers. Furthermore, PANI has very low solubility and its solution has insufficient viscosity and elasticity for processing. Even after doping, the PANI emeraldine salts do not have high enough solubility and they are still in dispersion state in solvents [9,11,12,13]. Preparing pure pristine PANI conductive textiles via conventional fiber spinning methods always encounters challenges. Current processing techniques to circumvent the challenges include coating on fibers/fabrics by in-situ polymerization and fiber spinning using conventional polymers as processing aids. Nanofiber mats (polyamide and poly(methyl methacrylate)), woven fabrics (polyimide and polyamide), and microfiber filaments (cellulose, chitosan, polyethylene and polyamide) have been reported as the substrates for coating [6,14,15,16,17,18,19]. The coated products had conductivity between 10^−3^ to 10^−1^ S/cm. The flexibility of the coated fabrics as well as the conductivity change with fabric bending and stretching were rarely reported. However, significant conductivity drop can be expected because the non-stretching and brittle nature of PANI makes the conductive paths break when the substrate bends and the interstices among yarns increase under fabric stretching. 

For PANI fiber spinning, conventional polymers as processing aids are commonly added to achieve the required spinnability. Electrospinning was the most common spinning method to prepare PANI fibers due to its versatility for comprehensive purposes and applications. Poly(ethylene oxide) (PEO), poly(lactic acid), polyvinylpyrrolidone, poly(methyl methacrylate), poly(vinylidene fluoride), and polystyrene have been frequently reported for blending with PANI [20,21,22,23,24,25,26,27,28,29,30,31,32,33,34,35,36,37,38,39,40,41,42]. Among these, PEO is the most used matrix polymer [20,21,22,23,43,44,45,46] because of its good spinnability and availability in wide range of molecular weight along with the nontoxic, fully biodegradable, and biocompatible traits [47]. PEO of high molecular weight allows its relatively low volumetric ratio to PANI in a spinnable blend solution in order to decrease the PANI percolation threshold and increase conductivity. Due to the difference in PANI concentration, matrix polymer used, fiber morphology and size, and conductivity measuring method, the resultant products had a wide range of electric conductivity reported from 1 S/cm to 10^–12^ S/cm. Additionally, the influence of PANI on the mechanical properties of the final products was seldom reported. 

In this study, we prepared novel electrospun bicomponent fibers containing PANI and PEO that were configured in a side-by-side fashion instead of blending by utilizing a barbed Y connector as the spinning nozzle. Pure doped PANI solution and pure PEO solution were fed into the two upper branches of the Y connector and combined to go through the lower branch which was connected to a hypodermic tube. The hypodermic tube was connected with a high voltage supply to charge the solutions for electrospinning. By adopting this design, PANI was in situ coated on the surface of PEO fibers during fiber production to combine coating and fiber spinning in one step with enhanced conductivity compared to blends. Furthermore, satisfactory mechanical property was demonstrated for the electrospun PANI/PEO mats using this method since PEO served as the substrate providing both flexibility and strength to the final mat products. The morphology, conductivity, mechanical properties, and relative resistivity during tensile test of the electrospun PANI/PEO mats prepared were evaluated and compared with PANI and PEO blends.

## 2. Materials and Methods 

### 2.1. Materials

Polyaniline (PANI, emerdine base, MW = 50,000 g/mol) were purchased form Sigma-Aldrich (St.Louis, MO, USA). Poly(ethylene oxide) (PEO, MW = 600,000 g/mol) and Camphorsulfonic acid (CSA, assay = 99%) were acquired from Acros (Waltham, MA, USA). Chloroform (CHCl_3_, assay = 99.8%) and N,N-Dimethylformamide (DMF, assay = 99.8%) were obtained from Macron Fine Chemicals (Radnor, PA, USA) and MilliporeSigma (Billerica, MA, USA), respectively. All chemicals were used as received without further purification.

### 2.2. Preparation of Polymer Solutions and Electrospinning

The doping and dissolving procedures were performed via mixing equimolar amount of CSA and PANI in CHCl_3_/DMF (3:1 volume ratio) solvent. The mixture was stirred for 16 hours and then ultra-sonicated (amplitude = 20% with a 1/4” Branson microtip) for 1.5 hours to form the PANI-CSA_CHCl_3_/DMF solutions. To avoid temperature rise during sonication, a salt water ice bath and intermittent sonication mode (40-secton on and 20-second off) were applied. The concentrations of PANI-CSA in the solutions were 1.5, 2.5, and 3.5 w/v%. Pure PEO solutions were prepared through dissolving PEO powder in CHCl_3_/DMF (3:1 volume ratio) mixture in concentrations of 3/4/5 w/v%. For PEO/PANI-CSA blend solutions, PEO powder was added into the prepared PANI-CSA solution and stirred overnight. 

**Figure 1:**
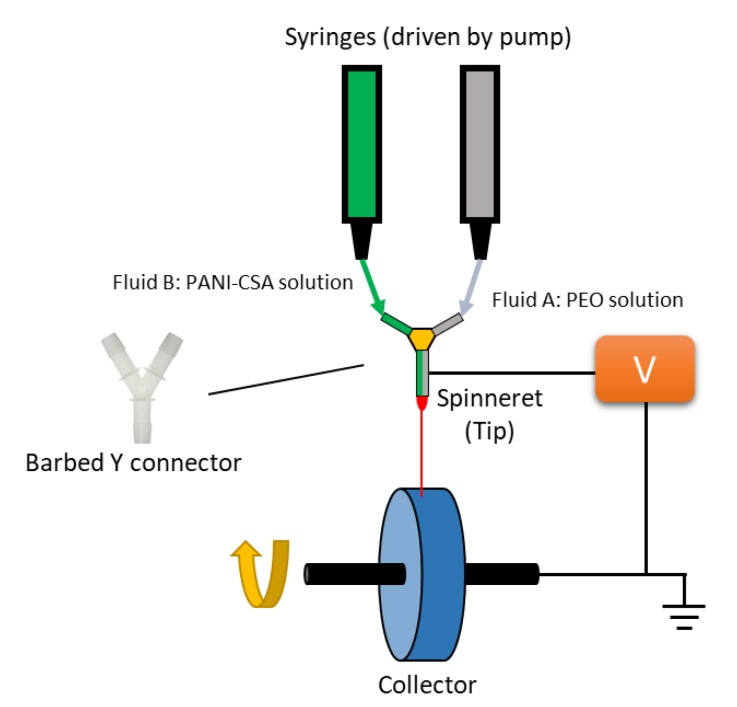
Schematic illusion of side-by-side electrospinning. PANI-CSA: Camphoric acid doped Polyaniline; PEO: polyethylene oxide.

Electrospinning was carried out with a system consisting of a high voltage supplier (Gamma high voltage), a syringe pump (Fusion 100T), and a grounded customized rotating disk as the collector for aligned fibers. There were two types of spinnerets used, i.e., a single 20-gauge needle for pure PEO and PEO/PANI blend fibers and a “side-by-side” barbed Y connector with 20-gauge stainless steel hypodermic tube for side-by-side fibers as sketched in Figure 1. Flow rate of the syringe pump was 0.45 mL/h, and applied voltage was 10 kV. The rotating speed was 600 rpm and the distance between the collector and spinneret was 15 cm. After collected, all fiber mats were placed in a well vented chamber overnight under room temperature (21 °C) before characterization.

### 2.3. Characterization

#### 2.3.1. Fiber Morphology

- Scanning electron microscopy (SEM)

Field emission scanning electron microscopy (Quanta 200F, Thermo Scientific-FEI, Waltham, MA, USA) was applied to examine fiber surface morphology. All samples were sputter-coated with 2.8 nm platinum before characterization. The electron field was 20 and 30 kV. Statistical analysis for fiber diameter was carried out using ImageJ software based on the measurement of 150 single fibers.

- Transmittance electron microcopy (TEM)

The field transmission electron microscope (Tecnai T20, Thermo Scientific-FEI, Waltham, MA, USA) was used to characterize PEO and PANI distribution inside fibers. TEM samples were prepared by collecting fibers on the surface of formvar/carbon coated nickel meshes during the electrospinning progress and dried in vacuum desiccator for 24 hours.

- Energy dispersive X-ray spectroscopy (EDS)

SEM (Tescan Vega3) with Energy Dispersive X-ray Spectroscopy (EDS) (Team EDS system, AMETEK EDAX, Middleboro, MA, USA) attachment was used for chemical content characterization of the electrospun nanofibers mats. All samples were sputter-coated with 15 nm thickness gold before characterization. The amp time was set at 7.68 µs.

#### 2.3.2. Tensile Test

Tensile properties of fiber mats (in the direction of fiber alignment) were evaluated on a universal testing machine (4466, Instron, Norwood, MA, USA) equipped with a 100 N load cell. The grip distance was 1.5 cm and the crosshead speed was maintained at 1.5 cm/min (100% strain per minute). Five replications for each sample were measured. All specimens were 1.0 cm wide and 4.0 cm long.

#### 2.3.3. Electrical Resistance and Conductivity 

A multimeter system, including a data acquisition/data logger switch unit (Agilent 34972A LXI, Keysight Technologies, CA, USA) and an armature multiplexer module (34901A, Keysight Technologies, Santa Rosa, CA, USA), was utilized for measuring electrical resistance. Relative resistance was measured using the same multimeter system during tensile testing. The probes of the multimeter were connected to the two grips of the tensile tester using copper adhesive tapes. Electrical conductivity was calculated based on resistance via Equation (1):

(1) $$\sigma =\frac{L}{R\times A}$$

where σ (S/cm) is the electrical conductivity of fiber mats, L (cm) is the distance between the two electrodes and A (cm^2^) is the cross-sectional area of fiber mats. 

#### 2.3.4. Thermogravimetric Analysis (TGA)

Thermal stability analysis of the fiber mats was performed using TGA (STAR e TGA/DSC 1 system, Mettler Toledo, Columbus, OH, USA) in nitrogen atmosphere. The scanning was conducted from room temperature to 600 °C with a heating rate of 10 °C/min. All specimens were dried in a desiccator for 24 hours prior to testing. 

#### 2.3.5. Viscosity

The viscosity of polymer solutions was determined using a universal viscometer (DV-E viscometer, AMETEK Brookfield, Middleboro, MA, USA) equipped with a thermostat jacket at room temperature. The shearing rate was 10 rpm. All results were based on five replicates.

#### 2.3.6. Fourier-Transform Infrared Spectroscopy (FTIR)

Chemical analysis was conducted using FTIR (Thermofisher Nicolet IS 50, Waltham, MA, USA) equipped with ATR configure. Samples were scanned between 500 and 4000 cm^–1^ with a resolution of 4 cm^–1^ and 64 scans. All results were based on three replicates. 

## 3. Result and Discussion

### 3.1. Solution Viscosity and Spinnability

For fiber spinning, polymer solution viscosity is a critical factor influencing solution spinnability and fiber morphologies. Both too low and too high viscosity can be challenging for the process. Solutions with too low viscosity have insufficient viscoelasticity to form a continuous polymer strand and beads often form along fibers; while excessive viscosity decreases the mobility of molecular chains and prevents them from being elongated into fibers [48]. Table 1 shows viscosities of the solutions with blended PEO and PANI at different concentrations in the CHCl_3_/DMF solvent. Pure PANI solutions with concentration from 1.5 to 3.5 w/v % had low viscosities that were close to that of the CHCl_3_/DMF solvent and they were not spinnable. Increasing PEO concentration from 3% to 5% enhanced solution viscosity from 555 to 2972 mPa·s when no PANI was presented. Blending PANI with PEO further increased the solution viscosity. It turned out when PANI was blended with 5% PEO, the solutions were too viscous to electrospin even with the lower PANI concentration of 1.5%. However, when solutions of 5% PEO and PANI were placed in the side-by-side manner, fibers could be produced. 

### 3.2. Morphology Study

**Figure 2:**
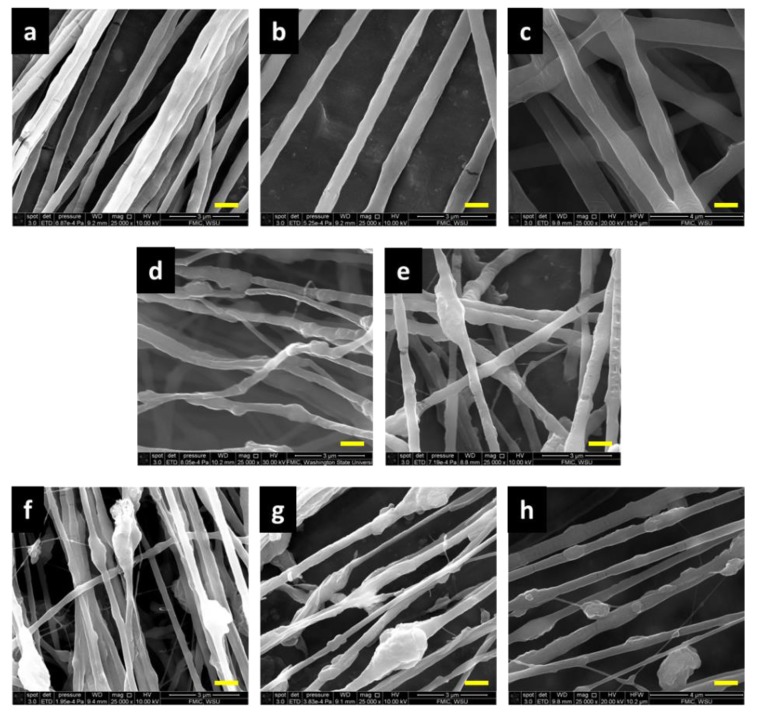
Scanning electron microscopy (SEM) images of fibers produced from solutions of pure PEO (**a**: 3%; **b**: 4%; **c**: 5%), blended PEO-PANI (**d**: 3%–2.5%; **e**: 4%–2.5%), and side-by-side PEO-PANI (**f**: 3%–2.5%; **g**: 4%–2.5%; **h**: 5%–2.5%). (Scale bar: 1 µm) (Magnification: 25,000×).

**Figure 3:**
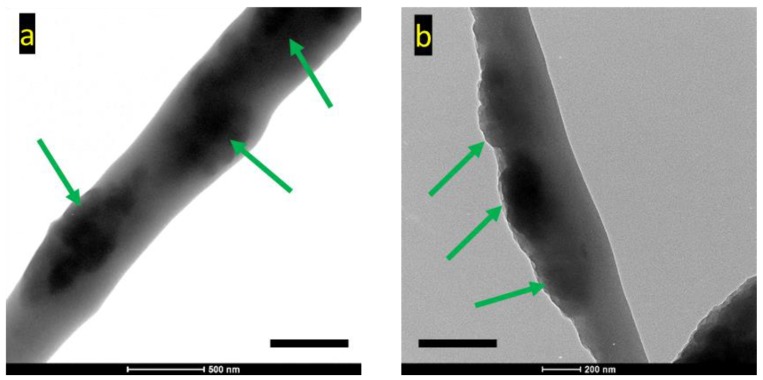
Transmittance electron microscopy (TEM) images of PEO-PANI (3%–2.5%) (**a**) Blended fiber, and (**b**) Side-by-side fiber (Scale bar: 500 nm). The arrows point to PANI-rich regions.

Figure 2 demonstrates the surface morphology of fibers produced from pure PEO, blended PEO/PANI, and side-by-side PEO/PANI. It can be seen that all fibers presented a general alignment along the rotating direction of the fiber collector. Pure PEO fibers without PANI were relatively uniform. Adding PANI reduced fiber uniformity and aggregated PANI formed beads and nodes along fibers. This was true for both blended and side-by-side fibers but more sever for the later. The reason could be attributed to the high surface tension of PANI (69 mN /m) [29]. During the fiber formation process when polymer jet traveled from the needle tip to the fiber collector, PANI agglomerated due to the low PEO and PANI interaction at the interface and the high surface tension of PANI. With the side-by-side spinning, the two solutions had limited contacting area and interaction time compared to the blended counterparts; this promoted the formation of PANI aggregates, which led to larger and more frequent PANI aggregates as shown in Figure 2f–h. In addition, the PANI aggregates were mostly buried in PEO matrix in the blends, but presented on one side of the side-by-side fibers as further demonstrated by the TEM images in Figure 3. The bright regions in the TEM images were PEO and the dark regions were PANI. As shown in Figure 3a, PANI was heterogeneously dispersed in the PEO matrix, which was also reported by others in the literature [29,36,49]. Although the blend fibers had PANI aggregates, their surface was still relatively smooth with PEO covering the aggregates even they were not uniform in dimeter along fiber length. Meanwhile, for the side-by-side fibers, images indicated that PANI was generally located unilaterally on the surface as demonstrated in Figure 3b. Therefore, the conductivity of the side-by-side fibers was expected to be higher than the blends, which was proven by the results shown in the later electrical conductivity section. 

**Figure 4:**
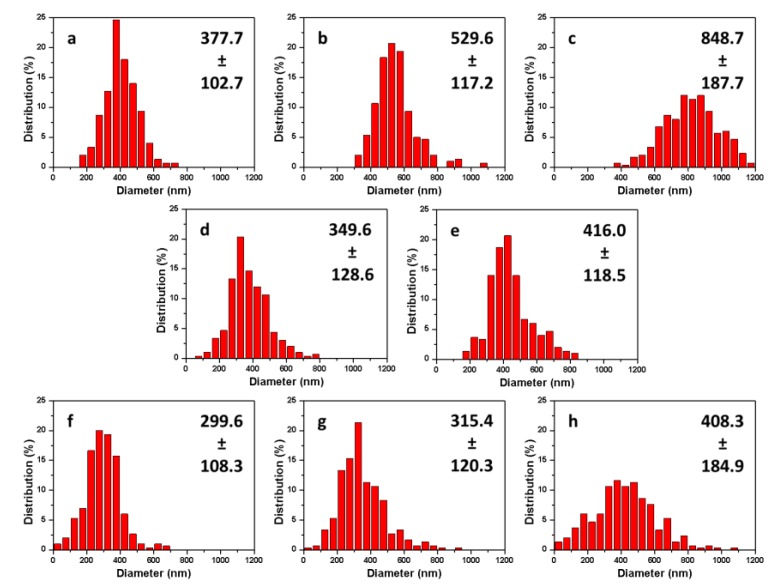
Diameter histograms of fibers produced from pure PEO (**a**: 3%; **b**: 4%; **c**:5%), blended PEO-PANI (**d**: 3%–2.5%; **e**: 4%–2.5%), and side-by-side PEO- PANI (**f**: 3%–2.5%; **g**: 4%–2.5%; **h**: 5%–2.5%). The insert data was fiber average diameter and standard deviation in nm.

In terms of fiber diameter, the concentration of PEO played a key role as expected and higher concentration led thicker fibers with and without PANI. Average fiber diameters increased from 378 to 849 nm for the pure PEO fibers and from 300 to 408 nm for the side-by-side fibers with PEO concentrations varying from 3% to 5% and PANI 2.5% as shown in Figure 4. When the PEO concentration increased to 5%, fibers displayed less uniformity with larger diameter standard deviation. Applying PANI resulted in a general tendency of reduced fiber diameter as well as increased diameter unevenness. This was because of the ionic nature of the doped PANI, i.e., PANI emeraldine salts [48,50]. The salts increased charge density of the spinning jet and both the speed and bending instability of the polymer jet were increased [36,51]. This result indicated that with the Y shape spinneret for the side-by-side spinning, solutions of PEO and PANI had limited contacting time from the top of the lower Y branch to the tip (less than 2 cm long) and the two solutions had mixed in a degree that was sufficient for them to overcome the jet instability and stay together to form fibers with both components. From Figure 3b, it can be seen that a small amount of PEO was mixed with PANI on one side of the fibers and there was no apparent boundary between the two components. This is unique and different from other published works, where distinct boundaries were observed for side-by-side Janus electrospun nanofibers [52,53,54,55,56]. The cohesiveness of the PEO and PANI components provided the fibers with good mechanical properties contributed by the PEO side as evidenced by the mechanical testing results presented later. In addition, durability of the PANI component on the side-by-side fibers should be good to withstand abrasion during use compared to direct coating of PANI on fabrics.

### 3.3. EDS for Nanofiber Chemical Composition Analysis

**Figure 5:**
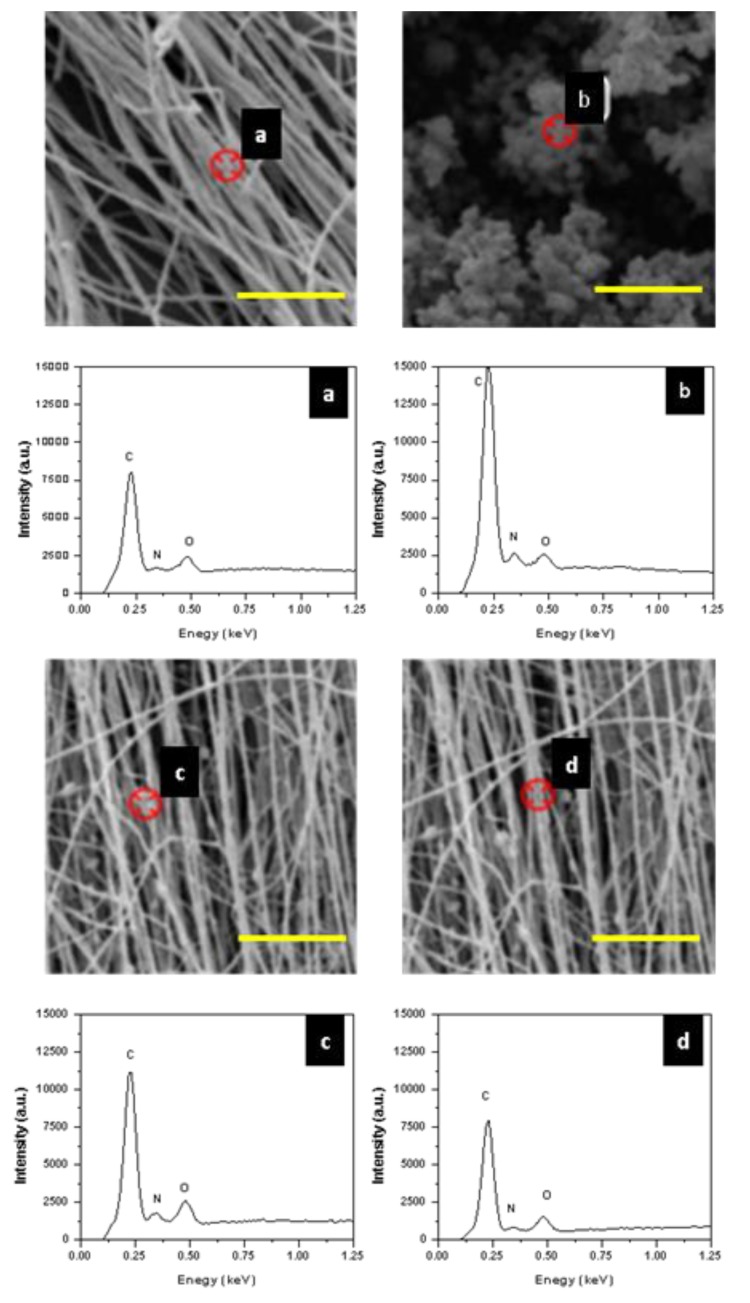
Energy-Dispersive X-ray Spectroscopy (EDS) surface analysis of (**a**) PEO 3%, (**b**) electrosprayed PANI-CSA, (**c,d**) 3%-2.5% side-by-side PEO-PANI fibers in knot and uniform areas, respectively. (Scale bar:10 µm).

The SEM-EDS point analysis was used to map/examine the presentation of PANI on fibers as illustrated in Figure 5. Neat PEO (Figure 5a) had a strong intensity of O atom that presented in the ether and hydroxyl groups on its backbones. A weak signal corresponding to N atom was also observed, suggesting the presence of minor DMF solvent remained in the elesctrospun nanofiber mat. In the electrosprayed PANI-CSA samples, both N atoms in PANI macromolecules and O atoms in CSA were detected (Figure 5b). For the side-by-side samples, results suggested that the knot position (Figure 5c) contained a high amount of PANI based on the relatively high intensity in N atoms compared to the intensity of O atoms. At the more uniform positions on the side-by-side fibers (Figure 5d), the relative N atom intensity was higher than that of pure PEO, indicating PANI was also presented. This suggested that the side-by-side fiber contained PANI along the length of the fiber forming a continuous conductive path not just at the aggregated sites.

### 3.4. TGA Analysis 

**Figure 6:**
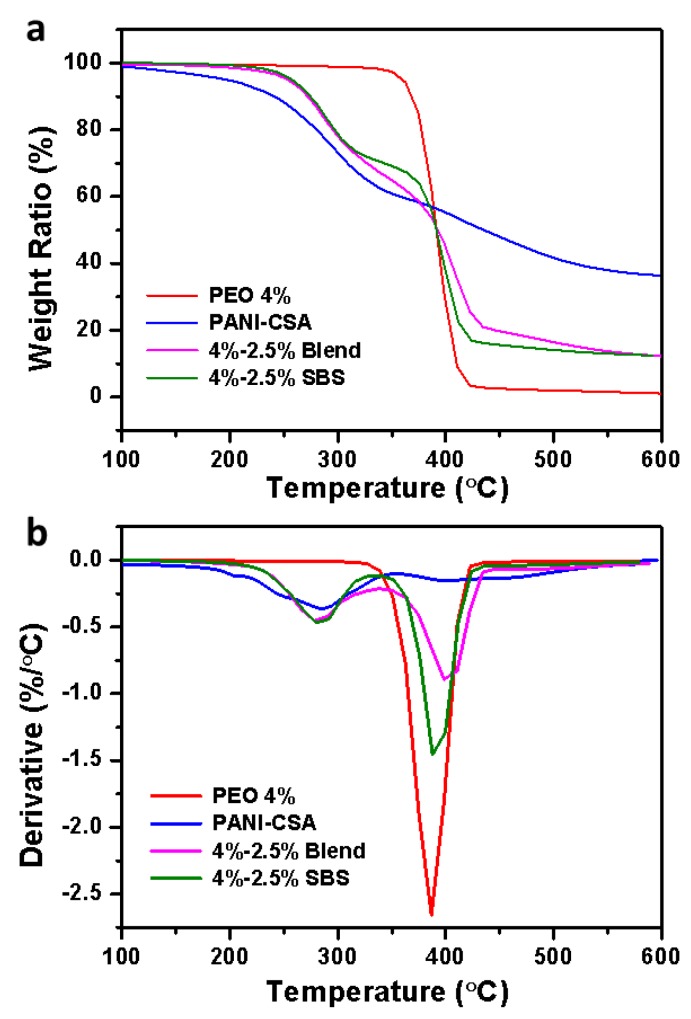
(**a**) Thermogravimetric analysis (TGA) and (**b**) derivative thermogravimetry (DTG) curves of neat PEO, PANI-CSA, blend and side-by-side PEO-PANI fibers (4%–2.5%).

Figure 6 depicted thermogravimetric behaviors of pure PEO, CSA doped PANI, 4%–2.5% blend and side-by-side fibers. Through both TGA and derivative thermogravimetry (DTG) curves, we could observe that pure PEO fibers displayed one-stage degradation from 360 to 430 °C while PANI-CSA followed a two-stage degradation process, i.e., doping acid (CSA) decomposition from approximately 190 to 330 °C and PANI degradation from 370 to 540 °C [57]. The decomposition of PANI was slow and the nonvolatile residue remained was high (~36.4 wt %). The blend and the side-by-side fibers had the same thermal degradation behavior below 310 °C. The onset degradation temperature for both was 210 °C because of the CSA. From 310 to 550 °C, the two fibers had different degradation paths. The side-by-side fibers showed distinct degradation behavior similar to PANI from 310 to 400 °C and distinct behavior similar to PEO from ~390 to 430 °C. The blend fibers exhibited a mixing effect of the two components. These thermal degradation results were consistent with the observed fiber morphology in that the PEO and PANI were more separated from each other in the side-by-side fibers compared to the blends. The residual contents of blend and side-by-side fibers were 12.1 and 12.4 wt %, respectively. The similar amount of residual proved that blend and side-by-side fibers had similar PEO and PANI composition. The hypothesis that unspinnable PANI could be spun into fibers with spinnable PEO using the side-by-side spinneret was proven. 

### 3.5. FTIR Analysis

**Figure 7:**
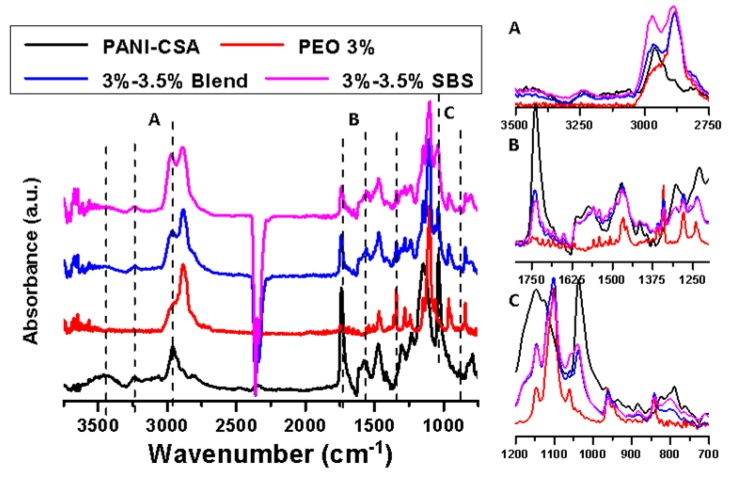
Fourier-transform infrared spectroscopy (FTIR) curves of PANI-CSA, neat PEO, PEO-PANI (3%–3.5%) blend and side-by-side fibers. The inserts (**A**, **B**, **C**) were enlarged images of three corresponding regions with the same baseline.

The FTIR results of PANI-CSA, pure PEO, 3%–3.5% blend and side-by-side fibers were illustrated in Figure 7. The PANI-CSA curve showed peaks at 780, 1020, 1170, 1214, 1300, 1485, 1590, 2900, 3250, and 3450 cm^−1^ [58,59,60,61], whereas pure PEO exhibited distinguishable peaks at 840, 1140, 1280, 1460, and 2840 cm^−1^ [62,63]. Taking a representative peak from each polymer as an example, unique N−H stretching of aromatic amines in PANI-CSA could be identified at 3450 cm^−1^; the peak at 1460 cm^−1^ for PEO was attributed to the C–O–C stretching in the backbone. Curves for blend and side-by-side nanofibers were very much similar and showed feature peaks that were combined from PEO and PANI-CSA and no new distinguishable peaks could be identified. This indicated that there was no clear chemical interaction between PANI and PEO. FTIR results also suggested that the unspinnable PANI had been successful spun into the nanofiber mats though our side-by-side electrospinning. Minor difference in peak height and width between blend and side-by-side existed, which might be related to their different fiber morphology. 

### 3.6. Electrical Conductivity 

The electrical conductivity of electrospun mats from side-by-side and blend fibers were illustrated in Table 2. Conductivity of all fibers was in the 10^−6^ to 10^−4^ S/cm range. The side-by-side fiber mats showed higher conductivity that was 8 to 15 times of that of the blend with the same PEO and PANI concentrations. Generally, increased PEO concentration reduced the mat conductivity because the relative PANI amount in the mat decreased. With a given PEO concentration, mat conductivity increased when PANI concentration raised from 1.5 to 2.5 and 3.5%. However, with all three PEO concentrations, the conductivity of 2.5 and 3.5% PANI was similar.

In the literature, electrospun PANI fibers had a wide range of conductivity, depending on many factors, such as the selection of matrix polymer, the volumetric ratio of the matrix polymer to doped PANI, and fiber morphologies. For instance, the conductivity of PANI and PEO blends was in the magnitude of 10^1^ S/cm to 10^−4^ S/cm in some studies [20,22], and the conductivity of PANI and PVP mats was lower than 10^−8^ S/cm reported by others [23,26,29,30,31,33,35,36,37,38,39,40,41,42]. It is also noteworthy that the conductivity testing method is a non-negligible factor. As Zhang and Rutledge [33] demonstrated, conductivity of individual fibers was generally 100~1000 times higher than that of the electrospun fiber mat. 

### 3.7. Tensile Properties 

**Figure 8:**
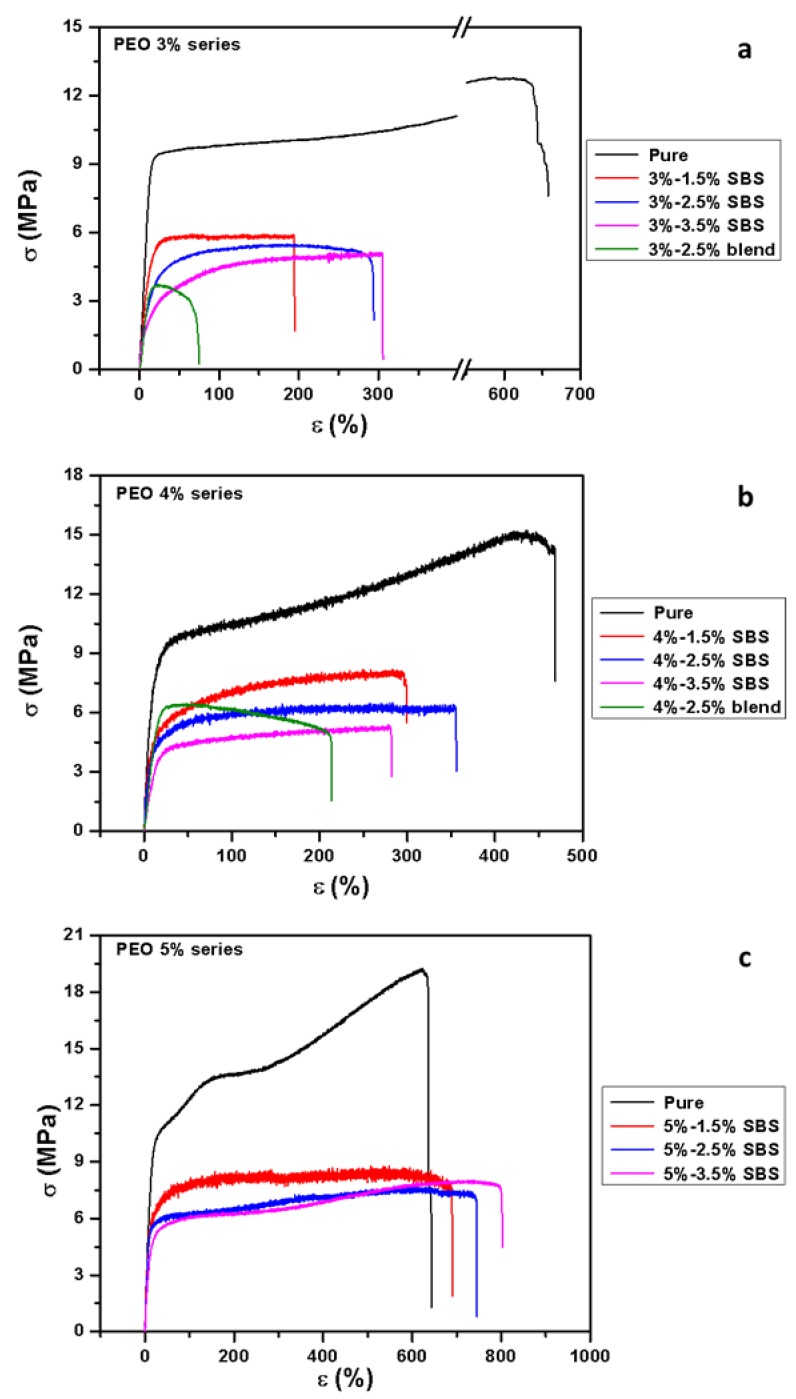
Stress-strain curves of electrospun fiber mats: (**a**) PEO 3% series; (**b**) PEO 4% series; (**c**) PEO 5% series.

Tensile properties of fiber mats measured in the direction of fiber alignment, including tensile modulus, tensile stress at 10% and 20% strains, elongation at break, and maximum strength, are shown in Table 3 and Figure 8. Pure PEO had excellent mechanical properties reflected by both strength (14.4 to 17.8 MPa) and elongation (490 to 618%). Increased PEO concentration enhanced fiber mat modulus, stress, and maximum strength. Adding PANI for both the blend and side-by-side fiber mats, modulus and strength were reduced as a function of the PANI content. This was because of the immiscible nature of the PANI and PEO resulting in poor interfacial adhesion between the two components without chemical bonding. Additionally, the doping acid CSA with soft molecular structure in a high content (20–50 wt % of overall fibers) might have plasticized the fibers and further reduced fiber stiffness and strength. Similar mechanical performance of PANI based blend electrospun mat was demonstrated by previous studies [27,64]. However, compared to the blends, the side-by-side fiber mats had improved mechanical properties, especially the elongation, which can be seen clearly from Figure 8a,b. With 3% PEO, blend fiber mat had an elongation of only 88% but those of side-by-side fiber mats were over 200%. The side-by-side PANI/PEO configuration had apparently overcome the fragile nature of PANI. The strength of PEO 3 series was strongly affected by the increased concentration of PANI, but PEO 5 series demonstrated less dependence due to the relative high PEO content. It needs to be pointed out here that the small amount of DMF solvent remained in the mats as demonstrated in the SEM-EDS results could have served as a plasticizer to increase fiber elasticity and decrease its strength. 

### 3.8. Relative Resistance during Tensile Stretching

**Figure 9:**
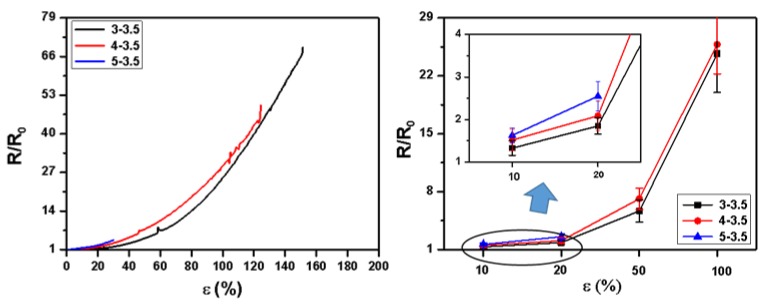
Relative electrical resistance of side-by-side fiber mats under different tensile strain.

For a tactile sensor (responsive to external physical interactions) that uses the strain-dependent resistivity change as the sensing mechanism, the relative electrical resistance is critical for its sensibility. Figure 9 illustrated the electrical resistance of the side-by-side fiber mats with 3.5% PANI. All tests were terminated when the mat resistivity reached the maximum measuring range of the multimeter used (200 MΩ). None of the samples tested broke at that point. Resistivity of the original fiber mats increased with the rising PEO concentration. Their resistivity was also increased with stretching as expected due to the breakage of conductive paths [65,66]. After being stretched, the strain under which the mat resistivity reached 200 MΩ were 30%, 125%, and 150% for 5%, 4%, and 3% PEO, respectively. Both 4% and 5% PEO-PANI side-by-side fiber mats displayed decent sensitivity and stretchability, which was superior to PANI blends. These properties are critical for applications, such as wearable sensors, artificial electronic skins and energy storage [3,65,67,68].

## 4. Conclusions

To overcome the poor spinnability of PANI, many studies blended PANI with conventional polymers as a processing aid to obtain electrospun composite fibers. In this study, we successfully utilized a barbed Y-connector as the spinneret to fabricate side-by-side electrospun PANI based fibers as evidenced by the fiber morphologies and chemical composition analysis. In these bicomponent fibers, PEO functioned as the substrate to carry PANI on one side of the fibers near their surface. Although no strong interactions between PEO and PANI was presented, microscopic images revealed some degree of mixing in the fibers with PEO covering PANI. This led to increased mechanical properties of the side-by-side fiber mats when compared to the blend fiber mats. The durability of the PANI component during real use to withstand abrasion is expected to be better than the coating method. TGA and FTIR results suggested that there was no significant loss of PANI during the electrospinning process. Although beads and nodes resulted from PANI aggregation were observed on the side-by-side fibers, PANI continuously presented along the length of fibers and conductive paths were formed. The roughly unilateral arrangement of the PANI component in the bicomponent fibers increased the conductivity of the side-by-side fiber mats. Given their relatively good flexibility and electrical conductivity, the side-by-side fiber mats demonstrated a great potential for flexible sensor applications, including smart wearable textiles. 

## Figures and Tables

**Table 1:** Viscosity of polymer solutions.

Viscosity (mPa·s)		PEO Content (w/v %)			
		0^1^	3^2^	4^2^	5^2^
PANI-CSA Content (w/v %)	0	1.07 ± 0.01 (solvent)	555 ± 7	1302 ± 26	2972 ± 42
	1.5	1.24 ± 0.01	630 ± 11	1585 ± 19	3952 ± 29
	2.5	1.33 ± 0.01	669 ± 12	1712 ± 11	4166 ± 21
	3.5	1.44 ± 0.02	703 ± 15	1894 ± 33	4753 ± 32

^1^ Speed: 100 rpm for PANI solution without PEO. ^2^ Speed: 12 rpm for solution contained PEO.

**Table 2:** Electrical conductivity (S/cm) for side-by-side and blends.

PEO-PANI	Blend	Side-by-Side
3%–1.5%	6.8–9.2 × 10^-6^	5.817.9 × 10^−5^
3%–2.5%	2.5–4.3 × 10^-5^	1.6–3.8 × 10^−4^
3%–3.5%	2.8–4.3 × 10^-5^	3.5–5.8 × 10^−4^
4%–1.5%	1.4–2.6 × 10^-6^	2.1–3.7 × 10^−5^
4%–2.5%	1.3–2.5 × 10^-5^	0.8–1.5 × 10^−4^
4%–3.5%	2.1–3.6 × 10^-5^	2.6–5.2 × 10^−4^
5%–1.5%	Not Spinnable	1.3–3.2 × 10^−6^
5%–2.5%		0.9–1.4 × 10^−4^
5%–3.5%		1.3–1.9 × 10^−4^

**Table 3:** General tensile properties of electrospun fiber mats of pure PEO, blend PEO/PANI, and side-by-side (SBS) PEO/PANI.

Code		Tensile modulus (MPa)	Stress at 10% strain (MPa)	Stress at 20% strain (MPa)	Elongation at break (%)	Maximum strength (MPa)
PEO 3%	Pure	63.8 ± 8.5	4.9 ± 1.0	7.0 ± 0.6	618 ± 149	14.4 ± 1.5
	3%–1.5% SBS	49.2 ± 3.0	3.7 ± 0.3	5.0 ± 0.3	202 ± 45	5.8 ± 0.3
	3%–2.5% SBS	38.6 ± 2.8	2.7 ± 0.2	3.3 ± 0.5	245 ± 79	4.9 ± 0.8
	3%–3.5% SBS	27.6 ± 6.5	2.2 ± 0.4	3.2 ± 0.6	292 ± 51	4.3 ± 0.6
	3%–2.5% blend	39.8 ± 6.2	2.9 ± 0.2	3.7 ± 0.2	88 ± 14	3.8 ± 0.2
PEO 4%	Pure	113.3 ± 13.2	6.3 ± 0.4	8.0 ± 0.3	490 ± 96	15.4 ± 1.2
	4%–1.5% SBS	91.3 ± 6.6	4.6 ± 0.2	6.5 ± 0.6	364 ± 75	8.3 ± 0.3
	4%–2.5% SBS	71.4 ±4.1	3.3 ± 0.3	4.2 ± 0.3	385 ± 78	6.7 ± 0.5
	4%–3.5% SBS	51.9 ± 6.9	2.7 ± 0.3	3.8 ± 0.3	305 ± 47	5.4 ± 0.4
	4%–2.5% blend	58.7 ± 6.2	4.2 ± 0.4	5.6 ± 0.5	201 ± 19	6.8 ± 0.3
PEO 5%	Pure	121.7 ± 20.8	6.7 ± 0.9	9.6 ± 0.6	604 ± 60	17.8 ± 2.5
	5%–1.5% SBS	86.3 ± 9.2	5.3 ± 0.3	6.1 ± 0.3	751 ± 62	8.2 ± 0.4
	5%12.5% SBS	78.7 ± 6.8	4.2 ± 0.2	5.5 ± 0.3	692 ± 53	7.8 ± 0.2
	5%–3.5% SBS	57.2 ± 7.6	3.8 ± 0.3	4.6 ± 0.4	721 ± 117	7.9 ± 0.6
	5%–2.5% blend	Not Spinnable				
